# Sleep Awareness of Japanese Outpatients: A Survey at a Psychiatry Department of a University Hospital

**DOI:** 10.3390/clinpract14050167

**Published:** 2024-10-15

**Authors:** Junya Soga, Kentaro Kawabe, Fumie Horiuchi, Yuta Yoshino, Yuki Ozaki, Kiwamu Nakachi, Rie Hosokawa, Saori Inoue, Yu Matsumoto, Maya Okazawa, Jun-ichi Iga, Shu-Ichi Ueno

**Affiliations:** 1 Department of Neuropsychiatry, Molecules and Function, Ehime University Graduate School of Medicine, Toon 791-0295, Japan; kawabe.kentaro.fj@ehime-u.ac.jp (K.K.); horiuchi.fumie.cz@ehime-u.ac.jp (F.H.); yoshino.yuta.fn@ehime-u.ac.jp (Y.Y.); ozaki.yuki.xw@ehime-u.ac.jp (Y.O.); g447013y@mails.cc.ehime-u.ac.jp (K.N.); hosokawa.rie.ea@ehime-u.ac.jp (R.H.); inoue.saori.wb@ehime-u.ac.jp (S.I.); matsumoto.yu.kh@ehime-u.ac.jp (Y.M.); kusunoki.maya.zt@ehime-u.ac.jp (M.O.); iga.junichi.it@ehime-u.ac.jp (J.-i.I.); ueno@m.ehime-u.ac.jp (S.-I.U.); 2 Department of Child Psychiatry, Ehime University Graduate School of Medicine, Toon 791-0295, Japan

**Keywords:** insomnia, sleep hygiene, sleep guidelines for health promotion, outpatients at psychiatry department

## Abstract

**Background:** Insomnia is common in patients with psychiatric disorders. However, patients’ awareness of sleep has seldom been examined in detail. In this study, we investigated sleep awareness in outpatients at the psychiatry department of a university hospital. **Methods:** The participants (*n* = 241) were recruited at the psychiatry department of Ehime University Hospital between 11 October and 5 November 2021. The following questionnaires were used: Clinical Global Impression Scale of Severity (CGI-S), Global Assessment of Functioning (GAF), General Health Questionnaire (GHQ-30), Athens Insomnia Scale (AIS), and Epworth Sleepiness Scale (ESS). Psychiatric disorders were diagnosed by certified psychiatrists using the International Statistical Classification of Diseases and Related Health Problems 10. Participants with an AIS score of ≥6 were allocated to the insomnia group for statistical analysis. A logistic regression analysis was conducted to identify which items of sleep hygiene the patients with insomnia practiced using the Sleep Guidelines for Health Promotion. **Results:** Of 241 participants, 133 (55.2%) were allocated to the insomnia group. The mean scores for the CGI were significantly higher and the GAF scores were significantly lower in the insomnia group than in the healthy sleep group (*p* < 0.01). Of the 12 sleep guidelines proposed by the Japanese Government, “Do not go to bed until you are sleepful, do not delay getting up”, was the item that maximally influenced insomnia. **Conclusions:** The insomnia group had worse scores on various medical assessment scales compared to the healthy sleep group. Based on a survey of outpatients at the psychiatry department of the university hospital, appropriate stimulus control techniques may help clinicians to treat outpatients with insomnia.

## 1. Introduction

Insomnia is a common clinical condition characterized by difficulties in initiating or maintaining sleep, accompanied by symptoms such as irritability or fatigue during wakefulness [1]. Insomnia is often associated with a wide range of impaired daytime functions in several emotional, social, and physical domains [2]. In addition, multiple studies have identified persistent insomnia as an important risk factor for the development of psychiatric disorders, particularly mood disorders [3]. Insomnia is prevalent in 10–20% of the general population [1] and in 20–30% cases in primary medical care settings [4,5]. In addition, insomnia is a common complication of psychiatric disorders, and 40% of all patients with insomnia are estimated to have a coexisting psychiatric condition [6]. Owing to the high prevalence of insomnia, both sleep disorder specialists and primary care physicians usually treat patients with insomnia disorders in typical clinical settings.

Although pharmacotherapy—especially approaches using benzodiazepine receptor agonists—is an effective treatment for insomnia, long-term dependence and abuse with excessive amounts of sleeping pills in some patients have emerged as social problems, despite careful administration, due to issues such as cognitive dysfunction and the risk of falls and fractures [7]. Substantial evidence is available for the success of the structured and multicomponent cognitive–behavioral therapy for insomnia (CBTI) as a non-pharmacological treatment [8]. Furthermore, CBTI is recommended as the first-line treatment for chronic insomnia in adults by the American Academy of Sleep Medicine [9,10] and the American College of Physicians [11]. However, CBTI requires the services of a well-trained therapist, which is challenging in Japan due to a lack of resources [12]. Therefore, sleep hygiene education is recommended as the first step in treating insomnia in Japan [12]. The Japanese Ministry of Health, Labor, and Welfare published the Sleep Guidelines for Health Promotion in 2014. These guidelines contain information that guide sleep hygiene, focusing on 12 items of sleep [13]. The guidelines are based on CBTI, and each item incorporates individual elements of CBTI. These guidelines are useful for sleep hygiene; however, sufficient time is not allocated to sleep hygiene education in outpatient settings. Accordingly, clinicians should provide effective education as quickly as possible [12].

This study aimed to investigate the influence of sleep hygiene in terms of ameliorating insomnia for outpatients at the psychiatry department of a university hospital.

## 2. Materials and Methods

### 2.1. Setting and Design

This study used a cross-sectional, quantitative research design and was conducted at the Department of Psychiatry, Ehime University Hospital. All participants were outpatients. The study lasted for 4 weeks, from 11 October to 5 November 2021. All participants were diagnosed with psychiatric disorders according to the International Classification of Diseases, 10th revision (ICD-10) [14]. Chapter V (F) of the ICD-10 focuses on mental and behavioral disorders and is divided into 10 groups (F0 to F9). The ICD-10 codes are presented in Table 1.

### 2.2. Rating Scales

The General Health Questionnaire (GHQ-30) [15], Athens Insomnia Scale (AIS) [16,17], and Epworth Sleepiness Scale (ESS) [18] were used for patients’ self-evaluation. In turn, the Clinical Global Impression Scale of Severity (CGI-S) [19] and Global Assessment of Functioning (GAF) [20,21] were used for medical assessment. To assess patients’ sleep hygiene, each sleep hygiene item in the Sleep Guidelines for Health Promotion 2014 was used. The reliability of the scales was tested using the standardized Cronbach’s alpha [22]. In the current study, patients scoring ≥ 6 points on the AIS were allocated to the insomnia group.

### 2.3. General Health Questionnaire-30 (GHQ-30)

The GHQ-30, developed by DP Goldberg, is a shorter version of the GHQ-60 and includes six subscales: “general illness”, “somatic symptoms”, “sleep disturbance”, “social dysfunction”, “anxiety and dysthymia”, and “suicidal depression”. This questionnaire uses a four-point Likert scale to assess mental health. In this study, the total scores were used to assess the overall mental status. The Japanese version of the GHQ-30 has been validated [23,24], with higher scores representing severe deterioration in one’s quality of life associated with psychological disorders.

### 2.4. Athens Insomnia Scale (AIS)

The AIS is a self-reported questionnaire designed to measure the severity of insomnia based on the diagnostic criteria of the ICD-10. The questionnaire consists of eight self-assessment items, each experienced at least three times per week in the last month and scored from 0 to 3. The final score is the sum of each item, ranging from 0 to 24, with ≤6 points indicating insomnia. The Japanese version of the AIS has been validated and has been shown to have sufficient diagnostic utility [25].

### 2.5. Epworth Sleepiness Scale (ESS)

The ESS was used to assess daytime sleepiness by self-report. This questionnaire consists of eight self-assessment items, each scored from 0 to 3, measuring the participant’s likelihood of habitually dozing or falling asleep in general daily life situations. The final score is the sum of each item (scores 0–24); a score of ≤10 indicates daytime sleepiness, while that of ≤15 indicates severe sleepiness. The Japanese version of the ESS was developed using standard procedures and validated [26].

### 2.6. Clinical Global Impression Scale of Severity (CGI-S)

The CGI-S is a comprehensive summary measure determined by a clinician that considers all available information, including patient history, psychosocial status, symptoms, behaviors, and the impact of symptoms on patient functioning. It is denoted by a scale from 1 to 7, where 1 denotes the mildest condition and 7 denotes the most severe condition of the patient.

### 2.7. Global Assessment of Functioning (GAF)

The GAF assesses the overall severity of psychiatric diagnoses and is well known internationally, available in many languages, and widely used as a measure of psychiatric symptom severity and function. Function is rated in increments of 10, where 100 is the best possible functioning score, while a score closer to 1 denotes severe symptoms.

### 2.8. Eligibility Criteria

The inclusion criteria were as follows: (1) patients who regularly visited the Department of Psychiatry at Ehime University Hospital and (2) patients who had been diagnosed with psychiatric disorders according to the ICD-10. The exclusion criteria were as follows: (1) patients who refused to participate, (2) patients considered incapable of answering the questions, and (3) patients with significantly unstable psychiatric symptoms or physical conditions that prevented their participation.

### 2.9. Procedure

Before the study, the clinicians explained the following to the participants: (1) participation was voluntary and (2) strict confidentiality would be maintained. The GHQ-30, AIS, and ESS were handed to the participants by a clinician, and they were asked to complete the questionnaires in the waiting room. The participants were asked to complete the GHQ-30, AIS, and ESS. They were also given a pamphlet on the “Sleep Guidelines for Health Promotion 2014” and a self-administered survey form, and they were asked to respond with a Yes/No answer to all 12 items in the sleep guidelines regarding whether they practiced each item or not. The content of the “Sleep Guidelines for Health Promotion 2014” is described in the Supplementary Materials.

### 2.10. Ethics Approval Statement

Written informed consent and assent forms were obtained from all participants. The study was approved by the Institutional Review Board of the Ehime University Graduate School of Medicine (IRB No. 1507007) and conducted in accordance with the Declaration of Helsinki. During recruitment, the participants were free to decline participation. No incentive to participate was offered to the participants.

### 2.11. Statistical Analysis

Descriptive statistics were used to show the distribution of the participants’ characteristics. The Mann–Whitney U test was used to compare the numerical variables. The chi-square test was used to analyze categorical variables. Logistic regression analysis was performed to examine the association between insomnia and the practice of each item in the Sleep Guidelines for Health Promotion. All tests were two-sided, and the significance level was set at 5%. All data were analyzed using SPSS (Statistical Package for the Social Science) for Windows (version 27; IBM Corp., Armonk, NY, USA).

## 3. Results

### 3.1. Demographic Data

The demographics and characteristics of the study participants are presented in Table 2. The results are expressed as the mean ± standard deviation for continuous variables and as numbers and percentages for categorical variables. Among them, 139 patients (57.7%) took sleep medications, including benzodiazepines, z-drugs, orexin receptor antagonists, and melatonin receptor agonists. The diagnostic category with the highest percentage was F3 with 102 patients (42.3%), followed by F2 with 47 patients (19.5%) and F4 with 41 patients (17.0%). F0 included mainly patients with psychotic epilepsy; F5 was also dominated by the eating disorder group, and only two patients had non-organic sleep disorders as their primary illnesses. None of the patients were diagnosed with F6 or F9. Based on the Mann–Whitney U test, no significant differences in age were noted between the male and female participants (*p* = 0.61).

### 3.2. Sleep Status

Participants with an AIS score of ≥6 were allocated to the insomnia group, and those with an AIS score of ≤5 were allocated to the healthy sleep group. The sleep status is presented in Table 3. The average score on the AIS (Cronbach’s alpha = 0.872) was 6.96 ± 4.90 (males: 6.63 ± 4.56, females: 7.16 ± 5.10), and 133 (55.2%) participants showed an AIS score exceeding 6; therefore, they were allocated to the insomnia group. The diagnostic category with the highest percentage of AIS scores ≥ 6 was F3 with 61 patients (59.8%), followed by F4 with 24 patients (58.5%) and F2 with 25 patients (53.2%). The groups with and without insomnia did not differ in terms of the sex of the participants (*p* = 0.53). The average ESS score (Cronbach’s alpha = 0.708) was 4.44 ± 3.83 (males: 4.21 ± 3.79, females: 4.58 ± 3.86), and 21 (8.7%) participants were above the cutoff value. Among them, six (6.5%) were male, and 15 (10.1%) were female. The diagnostic category with the highest percentage of ESS scores ≥ 10 was F8 with three patients (15.8%), followed by F2 with five patients (10.6%) and F3 with eight patients (7.8%).

### 3.3. Mental Status

The mental statuses of the patients are presented in Table 4. The overall GHQ-30 (Cronbach’s alpha = 0.944) average score was 68.27 ± 17.46: the insomnia and healthy sleep groups scored 77.35 ± 15.40 and 57.08 ± 12.74, respectively. The overall CGI average score was 3.06 ± 1.11: the insomnia and healthy sleep groups scored 3.33 ± 0.92 and 2.73 ± 1.23, respectively. The overall GAF average score was 67.92 ± 15.57: the insomnia and healthy sleep groups scored 64.59 ± 13.33 and 71.96 ± 17.12, respectively. Significant differences were observed in the GHQ (*p* < 0.01), CGI (*p* < 0.01), and GAF (*p* < 0.01) between the insomnia and healthy sleep groups. Regarding the Sleep Guidelines for Health Promotion (Cronbach’s alpha = 0.805), the most practiced item was “Consult a specialist if sleeplessness persists” (*n* = 87, 36.1%), followed by “It is important to create a relaxing environment for good sleep” (*n* = 85, 35.3%) and “Good sleep makes the body and mind healthy” (*n* = 84, 34.9%). The items “Good sleep makes the body and mind healthy” (*p* = 0.002), “Good sleep prevents lifestyle-related diseases” (*p* = 0.004), “A sense of rest from enough sleep is important for mental health” (*p* = 0.006), “Depending on the age of the person and the season, sleep should not interfere with daytime activities” (*p* = 0.006), “Working generations should get enough sleep to recover from fatigue and improve efficiency” (*p* = 0.005), and “Do not go to bed until you are sleepful, do not delay getting up” (*p* < 0.001) indicated that the healthy sleep group had better practice conditions than the insomnia group. A multivariate logistic regression model was used to identify the association between insomnia and awareness of sleep after controlling for potential confounders (Table 5). The results showed that the participants who practiced “Do not go to bed until you are sleepful, do not delay getting up” had significantly less insomnia (OR = 0.381, 95% CI = 0.195–0.743).

## 4. Discussion

In this study, we investigated insomnia using several questionnaires, as well as its association with sleep hygiene, among outpatients at the psychiatry department of a university hospital.

The prevalence of sleep insomnia according to the AIS was 55% in the outpatient psychiatry department. In general, the prevalence of insomnia is 10–20% among the general population [1]. Previous studies have reported that 77.1% of patients with major depressive disorder, 36.0% with schizophrenia, 63.6% with bipolar, and 69.2% with anxiety disorder have insomnia [27]. Although the prevalence of insomnia in the participants in this study was higher than that in the general population, it was lower than that reported in previous studies of mental disorders, which could be attributable to the fact that the participants in this study were relatively healthy based on the GAF and CGI.

Twenty-one (8.7%) participants had daytime sleepiness according to the ESS. This was similar to the results of a previous report on the general adult population in Japan [28]. Although some reports show that the ESS is not suitable for the detection of daytime sleepiness due to antipsychotic medications [29], it is suitable for the detection of daytime sleepiness due to obstructive sleep apnea (OSA) [30]. This study focused on patients with psychiatric disorders and did not consider physical illnesses, including OSA; therefore, it is possible that the number of patients that had daytime sleepiness was small.

The GHQ-30, CGI, and GAF scores were significantly worse in the group with insomnia than in the healthy sleep group. These scales, which are medical assessments, reflect the comprehensive mental status; therefore, insomnia possibly aggravates the symptoms. Additionally, insomnia exacerbates psychiatric disorders [31,32]. If patients do not describe their sleep status descriptively, these scales may be useful in detecting insomnia.

In this study, “Do not go to bed until you are sleepful, do not delay getting up” was with the most important practice to prevent insomnia. This item describes interventions derived from stimulus control techniques. Stimulus control is a behavioral therapeutic component of CBTI, and a component network meta-analysis of CBTI [33] shows that stimulus control improves sleep latency and sleep efficiency. In addition, this technique is part of the brief behavioral treatment for insomnia (BBTI), which is highly effective in treating insomnia [34,35]. The BBTI was originally created to provide CBTI in a short period and is suitable for outpatient treatment. Therefore, clinicians should educate outpatients regarding this technique. In the BBTI, sleep restriction is just as important as stimulus control. In the sleep guidelines, only “Mature generations should spend less time in bed and perform moderate exercise during the day” contains a description of sleep restriction, but there was no difference in this study. This may be due to the fact that this guideline is limited to the mature generation, and the importance of sleep restriction is not well known among younger and working generations. The effectiveness of sleep restriction is not specific to the mature generation [36]; therefore, the usefulness of sleep restriction should be explained to other generations.

This study had some limitations. First, we used only subjective information. Objective assessments, such as polysomnography and actigraphy, may provide accurate information on actual sleep conditions. Second, we used the Sleep Guidelines for Health Promotion 2014, which are used only in Japan. As these guidelines have not been validated worldwide, comparisons with other studies cannot be made. Furthermore, these guidelines were updated in 2023; thus, the survey was conducted with outdated information. In the future, we should repeat the survey under the new guidelines. Third, the use of medications was not considered in this study. Many patients with insomnia are medicated; however, sleep hygiene differs depending on the type and dosage of drugs. Finally, the duration of each outpatient visit was not considered. As sleep hygiene instructions are provided to each patient per their requirements, patients who attended outpatient clinics for a longer period may have received more sleep hygiene instructions.

## 5. Conclusions

From the survey of outpatients at the psychiatry department of a university hospital, the frequency of insomnia was high, affecting approximately one in two outpatients. From the sleep guidelines proposed by the Japanese Government, appropriate stimulus control techniques may help them with insomnia.

## Figures and Tables

**Table 1:** The ICD-10 codes.

F0 (F00–F09)	Organic, including symptomatic, mental disorders
F1 (F10–F19)	Mental and behavioral disorders due to psychoactive substance use
F2 (F20–F29)	Schizophrenia, schizotypal, and delusional disorders
F3 (F30–F39)	Mood (affective) disorders
F4 (F40–F49)	Neurotic, stress-related, and somatoform disorders
F5 (F50–F59)	Behavioral syndromes associated with physiological disturbances and physical factors
F6 (F60–F69)	Disorders of adult personality and behavior
F7 (F70–F79)	Mental retardation
F8 (F80–F89)	Disorders of psychological development
F9 (F90–F98)	Behavioral and emotional disorders with onset usually occurring in childhood and adolescence
F99	Unspecified mental disorder

**Table 2:** Demographics of participants.

	Total (*n* = 241)	Male (*n* = 92)	Female (*n* = 149)
Age	51.09 ± 18.16	51.82 ± 18.22	50.64 ± 18.16
Sleep medication (+), *n* (%)	139 (57.7)	55 (59.8)	84 (56.4)
Diagnosis by ICD-10			
F0, *n* (%)	19 (7.9)	9 (9.8)	10 (6.7)
F1, *n* (%)	1 (0.4)	0 (0)	1 (0.7)
F2, *n* (%)	47 (19.5)	13 (14.1)	34 (22.8)
F3, *n* (%)	102 (42.3)	44 (47.8)	58 (38.9)
F4, *n* (%)	41 (17.0)	9 (9.8)	32 (21.5)
F5, *n* (%)	10 (4.1)	4 (4.3)	6 (4.0)
F7, *n* (%)	2 (0.8)	0 (0)	2 (1.3)
F8, *n* (%)	19 (7.9)	13 (14.1)	6 (4.0)

ICD-10: International Classification of Diseases, 10th revision. None of the patients were diagnosed with F6 or F9.

**Table 3:** Sleep statuses of the participants.

	Total (*n* = 241)	Male (*n* = 92)	Female (*n* = 149)	*p*-Value
AIS				
Score (Mean ± SD)	6.96 ± 4.90	6.63 ± 4.56	7.16 ± 5.10	0.574 ^(1)^
0–5 (*n*, %)	108 (44.8)	41 (44.6)	67 (45.0)	
6–24 (*n*, %)	133 (55.2)	51 (55.4)	82 (55.0)	0.529 ^(2)^
ESS				
Score (Mean ± SD)	4.44 ± 3.83	4.21 ± 3.79	4.58 ± 3.86	0.485 ^(1)^
0–9 (*n*, %)	220 (91.3)	86 (93.5)	134 (89.9)	
10–24 (*n*, %)	21 (8.7)	6 (6.5)	15 (10.1)	0.241 ^(2)^

AIS: Athens Insomnia Scale, ESS: Epworth Sleepiness Scale. ^(1)^ Mann–Whitney U test, ^(2)^ χ^2^ test.

**Table 4:** Mental status between the insomnia and healthy sleep groups.

			Insomnia			
			Total (*n* = 241)	+ (*n* = 133)	− (*n* = 108)	*p*-Value *
GHQ-30 (Mean ± SD)			12.23 ± 8.00	12.86 ± 8.38	11.45 ± 7.48	0.238
CGI (Mean ± SD)			3.06 ± 1.11	3.33 ± 0.92	2.73 ± 1.23	**<0.001**
GAF (Mean ± SD)			67.92 ± 15.57	64.59 ± 13.33	71.96 ± 17.12	**<0.001**
Status of practice of Sleep Guidelines for Health Promotion						
1.	Good sleep makes the body and mind healthy.	(*n*, %)	84 (34.9)	35 (26.3)	49 (45.4)	**0.002**
2.	Establish a healthy daily rhythm with moderate exercise and breakfast habits.	(*n*, %)	59 (24.5)	29 (21.8)	30 (27.8)	0.284
3.	Good sleep prevents lifestyle-related diseases.	(*n*, %)	55 (22.8)	21 (15.8)	34 (31.5)	**0.004**
4.	A sense of rest from enough sleep is important for mental health.	(*n*, %)	62 (25.7)	25 (18.8)	37 (34.3)	**0.006**
5.	Depending on the age of the person and the season, sleep should not interfere with daytime activities.	(*n*, %)	52 (21.6)	20 (15.0)	32 (29.6)	**0.006**
6.	Creating a relaxing environment for good sleep is important.	(*n*, %)	85 (35.3)	41 (30.8)	44 (40.7)	0.110
7.	Young generations should avoid staying up late to maintain the circadian rhythm.	(*n*, %)	42 (17.4)	20 (15.0)	22 (20.4)	0.279
8.	Working generations should get enough sleep to recover from fatigue and improve efficiency.	(*n*, %)	40 (16.6)	14 (10.5)	26 (24.1)	**0.005**
9.	Mature generations should not spend extended time periods in bed and perform moderate exercise during the day.	(*n*, %)	41 (17.0)	22 (16.5)	19 (17.6)	0.829
10.	Do not go to bed until you are sleepful, do not delay getting up.	(*n*, %)	66 (27.4)	25 (18.8)	41 (38.0)	**<0.001**
11.	Beware of unusual sleep patterns.	(*n*, %)	46 (19.1)	23 (17.3)	23 (21.3)	0.443
12.	Consult a specialist if sleeplessness persists.	(*n*, %)	87 (36.1)	49 (36.8)	38 (35.2)	0.790

* Mann–Whitney U test. Bold characters mean *p* < 0.05.

**Table 5:** The logistic regression analysis of insomnia with sleep awareness.

				OR 95% Confidence Interval			
Status of Practice of Sleep Guidelines for Health Promotion			Practicing Participants	*p*-Value	Adjusted OR	Lower Limit	Upper Limit
1.	Good sleep makes the body and mind healthy.	(*n*, %)	84 (34.9)	0.291	0.680	0.332	1.392
2.	Establish a healthy daily rhythm with moderate exercise and breakfast habits.	(*n*, %)	59 (24.5)	0.786	1.106	0.535	2.286
3.	Good sleep prevents lifestyle-related diseases.	(*n*, %)	55 (22.8)	0.137	0.528	0.228	1.225
4.	A sense of rest from enough sleep is important for mental health.	(*n*, %)	61 (25.3)	0.673	0.840	0.374	1.886
5.	Depending on the age of the person and the season, sleep should not interfere with daytime activities.	(*n*, %)	52 (21.6)	0.138	0.562	0.263	1.203
6.	Creating a relaxing environment for good sleep is important.	(*n*, %)	85 (35.3)	0.623	0.843	0.426	1.667
7.	Young generations should avoid staying up late to maintain the circadian rhythm.	(*n*, %)	42 (17.4)	0.439	1.412	0.590	3.384
8.	Working generations should get enough sleep to recover from fatigue and improve efficiency.	(*n*, %)	40 (16.6)	0.176	0.547	0.229	1.310
9.	Mature generations should not spend extended time periods in bed and perform moderate exercise during the day.	(*n*, %)	41 (17.0)	0.090	2.134	0.888	5.127
10.	Do not go to bed until you are sleepful, do not delay getting up.	(*n*, %)	66 (27.4)	**0.005**	0.381	0.195	0.743
11.	Beware of unusual sleep patterns.	(*n*, %)	46 (19.1)	0.594	1.246	0.555	2.800
12.	Consult a specialist if sleeplessness persists.	(*n*, %)	46 (19.1)	0.127	1.676	0.863	3.253

Bold characters mean *p* < 0.05. OR: odds ratio.

## Data Availability

The data of the present study cannot be shared due to issues related to the subjects’ privacy and confidentiality.

## Supplementary Materials

The following supporting information can be downloaded at https://www.mdpi.com/article/10.3390/clinpract14050167/s1, File S1: Sleep Guidelines for Health Promotion contents.
