# Supplementary material for: Sleep Awareness of Japanese Outpatients: A Survey at a Psychiatry Department of a University Hospital
Source: Clin Pract. 2024 Oct 15;14(5):2116–24. doi: 10.3390/clinpract14050167 (PMC11506421; doi:10.3390/clinpract14050167)
Supplement: Supplementary file 1 [file clinpract-14-00167-s001.zip › clinpract-3220764-supplementary.pdf]

The contents of the Sleep Guidelines for Health Promotion are as follows.

Number 1: Good sleep makes the body and mind healthy.

Number 2: Establish a healthy daily rhythm with moderate exercise and breakfast habits.

Number 3: Good sleep prevents lifestyle-related diseases.

Number 4: A sense of rest from enough sleep is important for mental health.

Number 5: Depending on the age of the person and the season, sleep should not interfere with daytime activities.

Number 6: Creating a relaxing environment for good sleep is important.

Number 7: Young generations should avoid staying up late to maintain the circadian rhythm.

Number 8: Working generations should get enough sleep to recover from fatigue and improve efficiency.

Number 9: Mature generations should not spend extended time periods in bed and perform moderate exercise during the day.

Number 10: Do not go to bed until you are sleepful, do not delay getting up.

Number 11: Beware of unusual sleep patterns.

Number 12: Consult a specialist if sleeplessness persists.
